# S1P Provokes Tumor Lymphangiogenesis via Macrophage-Derived Mediators Such as IL-1*β* or Lipocalin-2

**DOI:** 10.1155/2017/7510496

**Published:** 2017-07-19

**Authors:** Shahzad N. Syed, Michaela Jung, Andreas Weigert, Bernhard Brüne

**Affiliations:** ^1^Institute of Biochemistry I, Faculty of Medicine, Goethe-University Frankfurt, 60590 Frankfurt, Germany; ^2^Project Group Translational Medicine and Pharmacology TMP, Fraunhofer Institute for Molecular Biology and Applied Ecology (IME), 60590 Frankfurt, Germany

## Abstract

A pleiotropic signaling lipid, sphingosine-1-phosphate (S1P), has been implicated in various pathophysiological processes supporting tumor growth and metastasis. However, there are only a few descriptive studies suggesting a role of S1P in tumor lymphangiogenesis, which is critical for tumor growth and dissemination. Corroborating own data, the literature suggests that apoptotic tumor cell-derived S1P alters the phenotype of tumor-associated macrophages (TAMs) to gain protumor functions. However, mechanistically, the role of TAM-induced lymphangiogenesis has only been poorly described, mostly linked to the production of lymphangiogenic factors such as vascular endothelial growth factor C (VEGF-C) and VEGF-D, or transdifferentiation into lymphatic endothelial cells. Recent findings highlight a rather underappreciated role of S1P in tumor lymphangiogenesis, referring to the production of interleukin-1*β* (IL-1*β*) and lipocalin-2 (LCN2) by a tumor-promoting macrophage phenotype. In this review, we aim to provide to the readers with the current understanding of the molecular mechanism how apoptotic cell-derived S1P triggers TAMs to promote lymphangiogenesis.

## 1. Introduction

One of the most striking features of the tumor microenvironment and during resolution of inflammation is the presence of apoptotic cells. For a tumor, in order to survive and metastasize, and for inflammation to resolve, there is the need for a drainage system to clear debris. Both a tumor and resolving inflammation produce several factors including sphingolipids that support the construction of a new drainage system, a process known as lymphangiogenesis. Lymphatics play an important role in clearing proteins, apoptotic cells, antigen-presenting cells, lymphocytes, and even tumor cells [1–5]. Although mechanistic aspects are only poorly understood, sufficient clinical and experimental evidence suggests that lymphangiogenesis provides a gateway to systemic metastasis [6–8]. In the tumor *micro-milieu* and during resolution of inflammation, alternatively activated macrophages (M2-polarized) play an important role. They are attracted and phenotypically reprogrammed by tumor and apoptotic cell-derived factors. It is generally accepted that apoptotic cell-derived sphingolipids, especially sphingosine-1-phosphate (S1P), influence the protumor and proresolving phenotype of macrophages [9–16]. We have demonstrated that the macrophage S1P receptor 1 (S1PR1) induced lipocalin-2 (LCN2) and caused NOD-like receptor pyrin domain-containing 3 (NLRP3) inflammasome activation and subsequent interleukin-1*β* (IL-1*β*) release. Both LCN2 and IL-1*β* are linked to lymphangiogenesis and tumor metastasis [17]. Despite these data, there is limited information on the role of S1P in lymphangiogenesis. Huang et al. reviewed the role of S1P in lymphangiogenesis from the perspective of lymphocyte trafficking [18]. Furthermore, in the context of tumor-induced lymphangiogenesis, S1P has been shown to provoke cell survival and migration [19, 20]. In this review, we present compelling evidence that S1P induces lymphangiogenesis by skewing macrophages to make them produce lymphangiogenic factors such as LCN2 and IL-1*β*.

## 2. Sphingosine-1-Phosphate Signaling

Sphingosine-1-phosphate (S1P), a bioactive lipid mediator, is produced by phosphorylation of sphingosine by two isoforms of sphingosine kinase (SPHK1 and SPHK2), whereas its degradation by S1P lyase generates (E)-2-hexadecenal and phosphoethanolamine [21]. S1P is also reversibly dephosphorylated by S1P phosphatase to regenerate sphingosine, and the level of which is additionally controlled by fluxes through de novo ceramide synthesis and the sphingosine salvage pathways, tilting the balance from S1P to sphingosine and ceramide [22]. S1P is associated with various biological processes such as survival, growth, migration, invasion, angiogenesis, vascular maturation, and immunity [15, 22–25], whereas sphingosine and ceramide, as S1P precursors, are linked to cell growth arrest and apoptosis [26]. Expectedly, enhanced S1P production has been implicated in various physiological and pathological processes including cancer and autoimmune inflammation [27, 28]. Intracellular S1P may act as a second messenger to trigger calcium release from the endoplasmic reticulum [29–31]. Moreover, there are reports on several intracellular targets, such as histone deacetylases (HDACs) and tumor necrosis factor- (TNF-) associated factor 2 (TRAF2). Intracellular S1P functions as a cofactor for E3 ubiquitin ligase activity of TRAF2 in nuclear factor-*κ*B (NK-*κ*B) signaling and also enhanced inhibitor of apoptosis 2- (cIAP2-) mediated K63-linked polyubiquitination of interferon regulatory factor-1, essential for IL-1-induced chemokine production and inflammation [15, 32, 33]. Intracellular S1P can be exported by several transporters, such as the ATP-binding cassette transporters ABCA1 [34], ABCC1 [35, 36], and ABCG2 [36] and spinster 2 (SPNS2) [37–39]. Interestingly, ABCC1 and ABCG2 were originally identified as multidrug-resistant genes [36] and correlated with bad prognosis in breast cancer [40]. S1P released from cells stimulates cell type-specific G protein-coupled receptors (GPCR), known as S1P receptors (S1PR1–S1PR5) [41, 42] in autocrine, paracrine, and/or endocrine fashion, which is termed as “inside-out signaling” [25, 43]. Based on resolution of the atomic structure of S1PR1 in complex with an antagonist, it was suggested that released S1P partitions into the plasma membrane to access S1PR1. The lateral movement of S1P within the plane of the lipid bilayer and between two transmembrane helices is used to access the binding pocket of the receptor [44]. Activation of these GPCRs by S1P results in their differential coupling to downstream targets such as Rac, Rho, or other enzymes (e.g., ERK-1/2, AKT, phospholipase C, and adenylyl cyclase) [43, 45, 46]. The combination of cell type-specific S1P receptor expression and differential coupling with G-proteins determines a broad range of biological functions attributed to S1P.

## 3. S1P and Lymphangiogenesis

The concordant involvement of S1P in angiogenesis and lymphangiogenesis is widely assumed in the literature. Nevertheless, until recently, major emphasis was on S1P and blood vessel formation. The phenotype of impaired vascular maturation as observed in *S1pr1*^−/−^ mice clearly suggested a role of S1PR1 in neovascularization [47, 48]. It has been demonstrated that *S1PR1* is upregulated in tumor vessels and its local knockdown suppressed vascular stabilization and angiogenesis as well as tumor growth in implanted Lewis lung carcinoma cells [49]. In vitro silencing of *S1PR1* in mouse endothelial cells inhibited cell migration, which is an early critical step in angiogenesis [49]. Moreover, S1P stimulated angiopoietin-2 (ANG2) secretion from lymph endothelial cells (LECs) much more potently than from blood vascular endothelial cells [50]. Given that ANG2 is required for lymphatic development [51, 52], S1P may act synergistically with ANG2 in lymphangiogenesis.

Recent evidence indicates that tumor cells can also induce lymph node (LN) lymphangiogenesis even before they metastasize and that metastatic tumor cells continue to induce lymphatic vessel growth within sentinel LNs, which is thought to promote their further metastatic dissemination [53]. LECs provide S1P in the cortical sinus of LNs for lymph node lymphangiogenesis [54]. Interestingly, a LEC-specific deletion of SPHK1 in SPHK2 knockout mice inhibited lymphatic vessel maturation [55]. MCF-7 tumor cell-specific overexpression of SPHK1 promoted microvessel density in the periphery of larger tumors with higher frequency in nude mice [56]. Conversely, downregulation of SPHK1 in cancer cells enhanced apoptosis and chemosensitivity, with subsequently reduced tumor growth [57]. Targeting S1P signaling by SPHK2 dysfunction significantly suppressed cancer development in the mouse model of colitis-associated cancer [58], whereas SPHK1 inhibition reduced peritumoral lymphatic density and LN metastases [20]. SPHK1 can be stimulated with VEGF, TNF-*α*, and S1P itself [59–61]. Thus, it is rational to assume that there is a feedforward loop, where S1P induces VEGF-C, which may increase S1P concentrations in the local microenvironment. Taken together, SPHK1 and S1P in LECs are required for the proper development of lymphatic vessels.

## 4. Macrophage Polarization and Their Interaction with Apoptotic Cells

Macrophages are often viewed as “sentinels” that reside in and/or patrol tissues in search for pathogens or dead cells, which refers to their known function as a “garbage disposal unit.” This view has changed dramatically over the last decades, and we now appreciate the huge diversity of these cells, their ability to profoundly adapt to their microenvironment and to perform unique local functions. As cells of the innate immune systems, their function goes far beyond host defense and removal of cellular debris and even these fundamental reactions are not any longer considered to be passive, rather evoking complex cell-cell interactions, with the release of a whole arsenal of communicating molecules. Different subpopulations of macrophages play essential roles in mounting immune responses during development and initiation as well as resolution of injury, chronic inflammation, and inflammation-driven cancer [62, 63]. Macrophages have a plastic phenotype with an overlapping spectrum of dynamic responses from classically activated (also known as M1) to alternatively activated (also known as M2). Classically activated macrophages are stimulated by lipopolysaccharide (LPS), interferon gamma (IFN-*γ*), and tumor necrosis factor-alpha (TNF-*α*) and provoke secretion of cytokines including IL-1, IL-6, IL-12, IL-23, and TNF-*α* and reactive nitrogen and oxygen intermediates (RNI, ROI) [64]. In contrast, anti-inflammatory stimuli such as IL-4, IL-13, IL-10, and glucocorticoid or immune complexes (IC) plus lipopolysaccharide (LPS) induce macrophages to an M2 phenotype. This type is characterized by a decreased production of inflammatory cytokines, increased production of anti-inflammatory cytokines (e.g., IL-10), and factors that mediate immunosuppression and tissue remodeling. These alternatively activated macrophages have been subgrouped. The M2a type is generated in response to IL-3 and IL-13, while M2b responds to immune complexes and toll-like receptor (TLR) activation. M2c macrophages represent deactivated macrophages that suppress proinflammatory cytokines, while the M2d type represents a regulatory macrophage [65] that is often grouped with tumor-associated macrophages (TAMs) [64, 66–68]. TAMs originate from circulating monocytes, which are recruited to the tumor microenvironment and reprogrammed by tumor-derived factors such as S1P, colony-stimulating factor-1, VEGF-A, and CC chemokine ligand 2 (CCL2). They also show a “smoldering” inflammatory phenotype that promotes cancer-related inflammation [69–72]. Key features of TAMs such as the production of tumor-promoting factors (e.g., PGE_2_, VEGF, EGF (epidermal growth factor), TGF-*β* (transforming growth factor-*β*), or MMP9 (matrix metalloprotease)), poor ROI production, high anti-inflammatory cytokine, and low proinflammatory cytokine production emerge as a consequence of macrophage interaction with apoptotic cells [72]. Mechanistically, the direct interaction of apoptotic cells with phagocytes via so-called eat-me signals or the production of apoptotic cell-derived soluble mediators that act on bystander cells accounts at least partly for the phenotype changes in TAMs.

The abundance of dying cells in tumors seems counterintuitive based on the common perception of cell death evasion being a tumor hallmark [73]. Although net growth certainly is a characteristic of the overall tumor population, it does not exclude the occurrence of cell death in a significant proportion of tumor cells as a result of multiple stress factors. In fact, apoptosis has originally been studied in neoplasms, and it was stated that the “spontaneous and continuous death of cells is an inherent property of malignant neoplasms” [74]. Apoptosis has been identified as the dominant form of cell demise in a number of malignancies based on morphological features such as nuclear condensation and the presence of apoptotic bodies. Controlled cell disintegration appears not to be just an epiphenomenon of tumor growth, since high apoptotic cell indices are linked to patient prognosis and metastasis [75, 76]. The tumor-promoting effects of apoptotic cells can be rationalized through their interaction with TAMs [75]. The communication is based on the concept that professional phagocytes are recruited to tissues with high rates of apoptosis to remove dying cells and their subsequent differentiation into a tumor-supporting cell type. Apoptotic cells produce a number of signals to instruct their own clearance (find-me signals). Find-me signals are molecules of diverse biochemical nature such as the lipids lysophosphatidylcholine (LPC) and S1P and the proteins fractalkine (CX_3_CL1), ribosomal protein S19, and EMAPII (endothelial monocyte-activating polypeptide 2) as well as nucleotides, for example, ATP and UTP [77]. Macrophage responses to apoptotic cells show some redundancy when comparing eat-me signals versus released soluble factors. This may work as a backup system to limit immune activation when apoptotic cell removal is attenuated [78].

Moreover, apoptotic tumor cell-released S1P induced activation of HIF-1*α*, which caused VEGF production provoking angiogenesis, which is a prerequisite for metastasis [79, 80]. Furthermore, S1P induced the formation of prostaglandin PGE_2_, thereby limiting CD80 expression in macrophages to inhibit antitumor immunity and to promote angiogenesis [81–83]. Tumor-derived S1P triggers S1PRs on macrophages and reprograms their phenotype towards tumor supportive, by inducing antiapoptotic signaling cascades to stabilize the antiapoptotic proteins Bcl-2 and Bcl-X_L_ [84]. In addition, there is increased IL-10 as well as IL-8 release, a higher arginase-1 activity to attenuate nitric oxide (NO) production, which in combination with decreased cytokine production marks alternative macrophage activation [14, 85]. S1P derived from apoptotic cells activated phosphoinositide 3-kinase (PI3K), extracellular signal-regulated kinase (ERK) 1/2, and Ca^2+^ signaling in primary human macrophages, thereby protecting them against TNF-*α*/cycloheximide-induced cell death [12]. Furthermore, S1P inhibited mitochondrial translocation of cytochrome-c and therefore activation of caspase-3 upon treatment with apoptosis-inducing stimuli [86]. S1P-mediated antiapoptotic responses are, however, not consistently linked to changes in the expression of antiapoptotic proteins, as the expression of Bcl-2 or Bcl-X_L_ was not seen in Jurkat T cells, U937 monocytes, or HL-60 cells [86]. Overall, apoptotic cells appearing as a consequence of tumor development support tumor progression through programming TAMs.

## 5. Macrophages in Lymphangiogenesis

A growing tumor relies on lymphatic vessels for the disposal of noxious antigens and removal of debris as well as a doorway to distal metastasis, whereas during resolution of inflammation lymphatics serves the purpose of draining debris. Inflammation is known to remodel the lymphatic network and to stimulate the growth of new lymphatics [6]. Tumor cells themselves or infiltrated immune cells of the tumor stroma, especially TAMs, either directly provide a conducive environment for lymphangiogenesis or indirectly generate prolymphangiogenic factors like VEGF-C, VEGF-D, and MMP9. However, it has been demonstrated that a TAM-mediated lymphangiogenesis usually forms abnormal and leaky lymphatic vessels, which facilitates cancer cells to metastasize to distal organs [87, 88]. Furthermore, TAM-derived VEGF-C or B cell-derived VEGF-A further suppresses antitumor immunity through local tolerance in LNs, either directly via VEGFR-2 signaling or by upregulating the lymphangiogenic factors VEGF-C and VEGF-D, which drives progression and metastasis [89, 90]. Interestingly, TAMs can also induce VEGF-C in tumor cells [91–94], thereby amplifying signal strength. Zhang et al. demonstrated in the Lewis lung carcinoma model that M2 macrophages induced VEGF-C expression in tumor cells [95]. Along these lines, depleting VEGFR-3^+^ TAMs with clodronate liposomes significantly reduced the secretion of VEGF-C and VEGF-D within tumors and concomitantly lowered lymphatic microvessel density [96]. Nevertheless, there are different ways how macrophages induce and regulate lymphangiogenesis. Indirect mechanisms comprise the induction of enzymes such as MMPs, plasmin, and urokinase plasminogen activator (uPA) that regulate matrix remodeling and growth factor activation. It has been shown that lymphatic vessel formation is controlled by uPA, MMP-2, and MMP-9, which facilitates LEC migration through collagen fibers, whereas plasmin has been reported to activate VEGF-C and VEGF-D [97, 98]. One of the most remarkably features of TAMs recently highlighted is their transdifferentiation into LECs [99, 100]. Evidence comes from studies colabeling macrophages with lymphatic endothelial cell markers such as LYVE-1 (lymphatic vessel endothelial hyaluronan receptor 1) and podoplanin. As such, CD11b^+^ macrophages were shown to form lymphatic-like structure in vitro in Matrigel that were positive for LYVE-1 and podoplanin [101]. Both murine and human TAMs can express a major lymphatic vessel marker, LYVE-1 [102, 103]. Hence, macrophage lymphatic vessel colocalization studies, especially in tumor sections, must be analyzed carefully to verify the results. Nevertheless, a relatively low percentage of macrophage transdifferentiation suggests that this process might be secondary to the dominant paracrine mechanisms of VEGFs.

**Figure 1:**
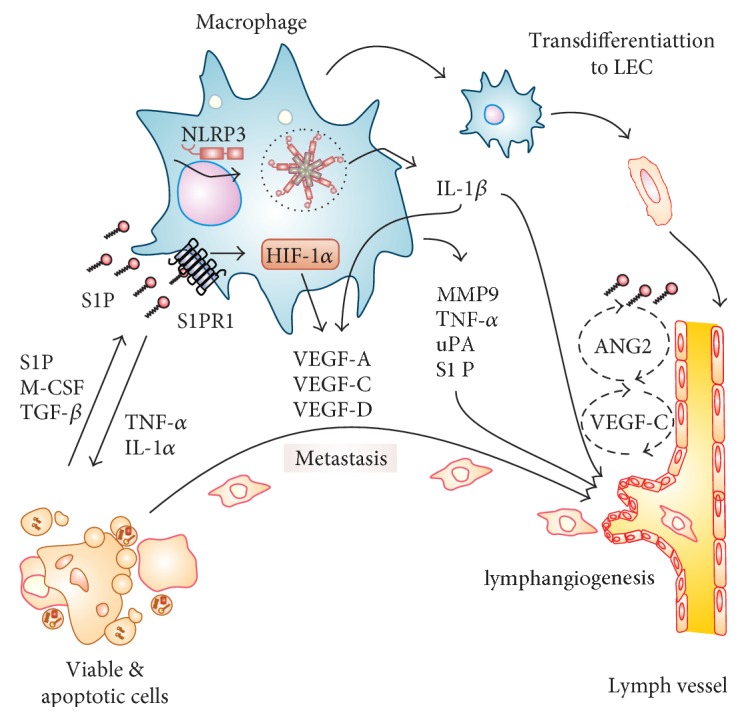
S1P-activated macrophages in lymphangiogenesis. Apoptotic cell-derived factors such as S1P, M-CSF, and TGF-*β* influence the macrophage phenotype. Triggering S1PR1 on macrophages causes NLRP3 inflammasome activation with the subsequent maturation of IL-1*β*. Released IL-1*β* provokes lymphangiogenesis in autocrine and paracrine manner by inducing VEGF-C in macrophages and endothelial cells. In macrophages, hypoxia-induced transcription factor HIF-1 controls the expression of VEGFs. Macrophages produce various factors such as MMP9, TNF-*α*, uPA, and IL-1*α*. These, together with VEGFs, induce lymphangiogenesis directly or by inducing VEGFs production by endothelial cells in multiple steps (discontinuous line). S1P also induces secretion of ANG2 from LECs (by unknown mechanisms, discontinuous line), to trigger lymphangiogenesis. In addition, macrophages may transdifferentiate to LECs. For details, see the text.

Lymphangiogenesis has spatial and temporal relationships with angiogenesis. Lymphatic microvascular networks are coordinated with the blood microvasculature to affect the local tissue fluid balance, tissue perfusion, and immune surveillance [104, 105]. Interestingly, Janus-faced macrophages have an intimate relationship with both blood and lymphatic endothelial cells, and as described below, macrophages play a bridging role at least in the context of cancer. As shown in Figure 1, TAMs also express the classic proangiogenic factor VEGF-A, which not only indirectly induces lymphangiogenesis by recruiting more macrophages [106] but also promotes proliferation and migration of VEGFR2^+^ LECs in vitro and induces sentinel LN lymphangiogenesis in a skin cancer model [107, 108]. Anti-VEGF-A treatment reduced both blood and lymphatic vascular densities, decreased VEGFR3 expression in LECs [106], and reduced metastasis in a breast cancer model [109]. Noteworthy, the above findings suggest that there is an overlapping involvement of VEGFs and their receptors in angiogenesis and lymphangiogenesis and conventional demarcation relating VEGF-A to angiogenesis and VEGF-C and VEGF-D to lymphangiogenesis may not explain the full spectrum of biological responses.

## 6. Macrophages and Lymphangiogenesis: The Inflammasome Liaison

Macrophages play a key role in sterile and smoldering inflammation, which is one of the hallmarks of cancer, by virtue of their ability to mount as well as to control inflammation [73]. In an inflammatory tumor microenvironment, macrophages can be primed and activated by a variety of damage-associated molecular patterns (DAMPs) such as ATP, HMGB1, and BD-2, which are produced by apoptotic and/or necrotic cells to foster, among others, IL-1*β* production. Secretion of IL-1*β* from primed macrophages depends upon the formation of an inflammasome, which is a large molecular scaffold containing cytosolic pattern recognition receptors, adaptor proteins, and caspase-1. The pattern recognition receptor NOD-like receptor pyrin domain-containing 3 (NLRP3) is well characterized and most relevant to sterile inflammation [110]. It is believed that the priming stimuli can include the activation of any receptor that causes activation of the transcription factor NF-*κ*B, such as ligands for IL-1R1, TLRs, and NLRs and the cytokine receptors TNFR1 and TNFR2 [111, 112]. The activation of NF-*κ*B is critical for upregulating the transcription of both pro-IL-1*β* and NLRP3, as pro-IL-1*β* is not constitutively expressed and basal levels of NLRP3 are inadequate for efficient inflammasome formation [113]. The second signal for inflammasome activation derives from the diverse group of agonists that trigger the specific activation of NLRP3 and assemble the inflammasome complex, which finally culminates in caspase-1 activation. These activators include both exogenous and endogenous molecules such as crystalline components (alum, silica, asbestos, and monosodium urate) that require phagocytosis for activation, ATP acting through its cell surface receptor P2X7R, pore-forming toxins, such as nigericin and potassium efflux, ROI formation, or cathepsin B release [114]. IL-1*β* is a key inflammatory cytokine found in many pathological conditions, triggering multiple downstream inflammatory pathways. It would be counterintuitive and deleterious for a tumor if TAMs produce IL-1*β* to mount an antitumor immunity. Rather, the sophisticated hijacking machinery of tumors turns the crisis into an opportunity for tuning the macrophage-derived IL-1*β* into a VEGF-C-inducing agent, thereby inducing lymphangiogenesis. TAM-derived IL-1*β* may work in autocrine and paracrine fashion to induce VEGF-C from macrophages, endothelial cells, or LECs.

Recent findings suggest that TAMs are attracted to and reside in hypoxic areas of tumor in a semaphorin 3A-dependent manner [115], where all the triggers for inflammasome expression/activation are around. It can be envisioned that inflammasome activation in hypoxic TAMs could be coupled with tumor-derived DAMPs such as sphingolipids. Luheshi et al. demonstrated that sphingosine, and to a smaller extend S1P, acted as a DAMP by inducing the NLRP3-inflammasome-dependent secretion of IL-1*β* from mouse peritoneal macrophages [116]. However, nonphysiological concentrations of S1P were used to demonstrate the effect, which calls for a validation of the primary finding in a more physiological setting. Subsequently, a study conducted in human U937 macrophages using sphingosine and synthetic SPHK1 substrates suggested that indeed sphingolipids induce the NLRP3 inflammasome in a cathepsin B-dependent manner, pointing to lysosomal destabilization in inflammasome assembly [117]. Macrophages seem to be quite evolved with respect to inflammasome activation and regulation. Recently, it has been observed that upon activation of caspase-1, oligomeric NLRP3 inflammasome particles were released from macrophages. These particles further enhanced caspase-1 activity extracellularly as well as after phagocytosis by surrounding macrophages as particulate danger signals [118].

It is not a mere coincidence that in response to inflammatory stimuli such as LPS or TNF-*α*, infiltrated macrophages enhance VEGF-C and VEGF-D expression, whereas LECs, in close vicinity, express higher *Prox-1* and NF-*κ*B to upregulate VEGFR3 expression and to coordinate macrophage inflammasome activation and VEGF-mediated lymphangiogenesis [119, 120]. The production of IL-1*β* by TAMs induced HIF-1*α* expression and the release of VEGF-A from tumors, even under normoxic conditions [121]. It is debated whether the action of VEGF-A on lymphangiogenesis is direct or indirectly mediated [122]. Mice overexpressing VEGF-A have enlarged lymphatic vessels due to induced lymphatic vessel remodeling [123]. Interestingly, chronic cutaneous inflammation induced lymphangiogenesis and lymphatic vessel hyperplasia in VEGF-A-overexpressing mice, but not in wild-type mice, suggesting that excess VEGF-A induces lymphangiogenesis [123].

Ji and colleagues showed that TAMs secreted VEGF-C following TNF-*α* stimulation and acted on LEC independent of VEGFR3, thereby questioning previously reported direct angiogenic and lymphangiogenic effects of TNF-*α* in Lewis lung carcinoma and ovarian cancer models [124]. Although not considered in the previous study, TNFR1 is known to activate NLRP3 inflammasome, thereby releasing IL-1*β*. IL-*β* directly activated LECs, via IL1R, to produce VEGF-C, thus, directly linking macrophage NLRP3 inflammasome activation with lymphangiogenesis. In line with these findings, our own studies now linked S1PR1 signaling in macrophages to produce IL-1*β*, which promoted lymphangiogenesis and metastasis. The genetic deletion of the S1PR1 in a subset of TAMs infiltrating murine PyMT breast tumors prevented pulmonary metastasis and drastically reduced tumor lymphangiogenesis. Attenuated lymphangiogenesis in the primary tumor became also evident in nonmetastasizing methylcholanthrene-induced fibrosarcomas. Cell sorting and transcriptome analysis of TAMs from both tumor entities revealed reduced expression of the inflammasome component *Nlrp3* in S1PR1-deficient TAMs, which correlated with decreased IL-1*β* levels in tumor tissue. Pharmacological interference at the level of S1PR1 in macrophages attenuated IL-1*β* production and lymphangiogenesis using a Matrigel plug assay in vivo and LEC tube formation in vitro. Apparently, S1PR1 in CD11b^hi^ CD206^+^ TAMs is a nonredundant mediator of tumor lymphangiogenesis. These data imply a so far unappreciated role of the NLRP3 inflammasome in regulating lymphangiogenesis in tumors following S1P production and support the emerging rationale of targeting S1PR1 in cancer therapy.

## 7. Macrophages and Lymphangiogenesis: The Lipocalin-2 Alliance

**Figure 2:**
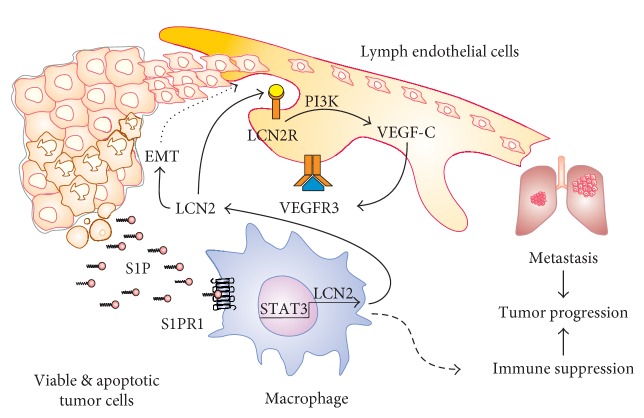
Macrophage-derived lipocalin-2 and lymphangiogenesis. Viable and apoptotic tumor cells produce S1P to stimulate the S1PR1 on macrophages. Downstream of S1PR1: there is activation of STAT3 to induce LCN2 mRNA and protein expression. LCN2 promotes EMT and drives lymphangiogenesis by activating its specific receptor, LCN2R, on lymph endothelial cells. The LCN2R activates PI3K, which adds to the production of VEGF-C, provoking a self-amplifying loop via VEGFR3. These signals promote metastasis and tumor progression. Macrophages add to immune suppression by distinct mechanisms. For details, see the text.

Naturally occurring or therapy-induced metabolic stress in tumor cells initiates autophagy, apoptosis, and/or necrosis, which in turn generates polarizing signals towards macrophages as indicated above. In established tumors, sustained apoptosis is a characteristic feature, which promotes tumor growth and progression. This can be interpreted as the tumor-exploiting apoptotic cell-derived signals that under physiological conditions, contribute to, for example, wound healing [9, 83, 125–127] to sustain its development. These considerations support the view of tumors as “wounds that do not heal” [128]. Given the importance of macrophages in the tumor microenvironment and their role in supporting several of the hallmarks of cancer, a signaling axis of sphingolipids, particularly S1P, acting on macrophages and thereby triggering a lymphangiogenic response became apparent. In cancer, lymphangiogenesis increases the risk of tumor cell migration to draining LNs and distal organ metastasis. We [17] used the protein kinase inhibitor staurosporine to induce apoptosis in MCF-7 breast cancer cells, prepared the conditioned medium from these dying cells, and subsequently added it to human macrophages. In turn, macrophages started to produce large mRNA and protein amounts of the siderophore-binding protein LCN2 in a manner partially demanding the signal transducer and activator of transcription 3 (STAT3). See Figure 2 for a schematic overview.

A role of STAT3 in LCN2 production was proven by pharmacological interference and LCN2 promoter reporter analysis with the full-length wild-type promoter or carrying a mutated STAT3-binding site. Conditional gene silencing confirmed the identity of the molecule produced in apoptotic tumor cells, responsible for the induction of LCN2 in macrophages, as the lipid S1P. A second step, using knockdown human macrophages or cells from conditional knockout bone marrow-derived macrophages from mice, identified the S1PR1 to transmit the signal into macrophages. Macrophage-derived LCN2 caused the sprouting and differentiation of mouse embryonic stem cells towards LYVE-1/podoplanin-positive cells and stimulated migration as well as proliferation of lymph endothelial cells (LECs). Mechanistically, LCN2 bound to its receptor (SCL22A17) on LECs drives VEGF-C protein production and secretion, which signaled in an autocrine manner via the VEGFR3 to promote proliferation. In vivo mammary tumor lung metastasis in MMTV-PyMT mice was impaired, with significantly attenuated lymph vessel formation in the primary tumor. Moreover, wild-type and LCN2 knockout tumor cell transplantation experiments pointed to the tumor stroma in generating LCN2 in promoting lymphangiogenesis. The release of LCN2 from macrophages also induced an epithelial-mesenchymal transition program in MCF-7 breast cancer cells and enhanced local migration as well as invasion of tumor cells into the extracellular matrix using a three-dimensional spheroid model [129]. Moreover, LCN2 deficiency attenuated tumor development in a xenograft model including lung metastasis when inoculating MCF-7 cells pretreated with supernatants from wild-type and LCN2-knockdown macrophages. These data underscore the significance of stroma-derived LCN2 on tumor cell dissemination, lymphangiogenesis, and metastatic growth, which points to a so far unappreciated S1P-LCN2 axis in the tumor microenvironment. Adding to the concept of tumor as wounds that do not heal, it was reported that LCN2 from macrophages exposed to apoptotic cells and thus, linked to S1P signaling via S1PR3, supported the proliferation and healing of renal epithelium, once inflammatory conditions were terminated [9].

Lipocalin-2 is a 25 kDa glycoprotein of the lipocalin superfamily [130, 131] that plays a pivotal role during, for example, bacterial infections [132] and cancer [133]. LCN2 displays pleiotropic functions that range from managing endogenous iron demand necessary for tumor cell replication, proliferation, survival, cellular differentiation, and migration, rendering LCN2 a putative mediator of tumor development [134]. Several studies indicated that LCN2 expression correlates with poor prognosis, especially in breast cancer [135] and serves as a prognostic and diagnostic marker, as elevated LCN2 is found in the urine of cancer patients. Previously, it was shown that LCN2 promotes lung metastasis of murine breast cancer cells in vitro and in vivo after injection of LCN2-overexpressing 4T1 cells [136] with the notion that LCN2 is linked to the early events of tumor metastasis. Mechanistically, this could be explained by stabilizing gelatinase B (MMP-9), thereby allowing enhanced degradation of the extracellular matrix and tumor cell dissemination [137, 138]. Taking into account that the tumor-promoting TAM phenotype also releases MMP-9, and its coformation with LCN2 may be well suited to promote cancer progression.

Over the last decade, our view on the role of innate immune cells in shaping the tumor microenvironment progressed significantly, now appreciating their role in promoting the hallmarks of cancer. Interestingly, players such as LCN2, involved in a protective response against microbial pathogens, are now recognized to pave the way for lymphangiogenesis and metastasis. These findings emphasize the parallelism of pathogen defence and cancer promotion linked to innate immune responses. Expanding on these considerations, inflammation, that is, tumor-associated inflammation, is recognized to contribute to all stages of tumor development [73].

## 8. Concluding Remarks

The interaction of macrophages with apoptotic cells promotes the release of factors such as S1P that are relevant not only for prognosis but also for intervention. There are tumor entities such as breast cancer having poor prognostic association with lymphangiogenesis, while others such as colorectal cancer showing a good correlative index and thus, may represent the extreme influential impact of S1P. By extrapolating the current understanding of the role of the macrophage S1P-S1PR1 axis in provoking lymphangiogenesis, it would be interesting to see whether conditions linking lymphangiogenesis and tumor progression will benefit from interfering with S1PR1 and/or its downstream signaling and thus, blocking the transforming capacity of TAMs. On the other side, it should be explored whether conditions that would benefit from effective lymphatic drainage, such as resolution of inflammation, gain a healing advantage from activating macrophage S1PR1. Along these lines, it has been demonstrated that a VEGF-C-dependent increases in lymphangiogenesis relieved the severity of acute skin inflammation and edema observed in an oxazolone-induced delayed type hypersensitivity reaction and ultraviolet B irradiation model [139]. However, one should be careful in generalizing the concept of lymphatics and resolution of inflammation since studies suggest that inhibition of the VEGF-C/VEGFR-2 axis suppresses lymphangiogenesis, rather than inducing it [122, 140, 141]. In addition, VEGFR-3 inhibition relieved the severity of inflammatory symptoms in rheumatoid arthritis and LPS-induced acute inflammation models [122, 142]. The dichotomy of macrophage S1PR signaling and the role of VEFG-A in both angiogenesis and lymphangiogenesis encourage further studies to probe the role of macrophages in unison with both types of vasculature.

The emerging role of apoptotic cell-derived S1P in triggering macrophage effector functions, especially the axis of NLRP3 inflammasome activation, IL-1*β* release, and lymphangiogenesis, as well as the S1P-LCN2 connection in the tumor microenvironment may pave a way for a new drug target discovery in cancer and inflammation.
